# Heterogeneous Nucleation in Protein Crystallization

**DOI:** 10.3390/biomimetics8010068

**Published:** 2023-02-06

**Authors:** Hao Liu, Yue Zhao, Jing Sun

**Affiliations:** 1 Key Laboratory of Biobased Polymer Materials, College of Polymer Science and Engineering, Qingdao University of Science and Technology, Qingdao 266042, China; 2 State Key Laboratory of Supramolecular Structure and Materials, College of Chemistry, Jilin University, Changchun 130012, China

**Keywords:** protein crystallization, crystallography, heterogeneous nucleation, crystallization strategy

## Abstract

Protein crystallization was first discovered in the nineteenth century and has been studied for nearly 200 years. Protein crystallization technology has recently been widely used in many fields, such as drug purification and protein structure analysis. The key to successful crystallization of proteins is the nucleation in the protein solution, which can be influenced by many factors, such as the precipitating agent, temperature, solution concentration, pH, etc., among which the role of the precipitating agent is extremely important. In this regard, we summarize the nucleation theory of protein crystallization, including classical nucleation theory, two-step nucleation theory, and heterogeneous nucleation theory. We focus on a variety of efficient heterogeneous nucleating agents and crystallization methods as well. The application of protein crystals in crystallography and biopharmaceutical fields is further discussed. Finally, the bottleneck of protein crystallization and the prospect of future technology development are reviewed.

## 1. Introduction

Proteins are biological macromolecules composed of one or more long chains of amino acid residues [1]. In the normal functions of an organism, such as metabolic catalysis, stimulation responses require the participation of proteins to function properly. The special biological function of proteins mainly comes from their special three-dimensional arrangement of amino acid residues in the active region. Since protein crystals were first observed in blood in 1840 [2], protein crystallization technology has undergone considerable developments and has been widely used in many fields. With the development of X-ray diffraction, it has become the core technology for analyzing protein crystal structure, which is the most effective means to determine the three-dimensional structure of proteins so far [3,4,5]. Compared with small molecular crystals, macromolecular protein crystals are smaller in size, less stable, easier to disintegrate, and not easy to subject to X-ray diffraction. The size and quality of the protein crystal will greatly affect the collection of X-ray diffraction data [6]. Obtaining high-quality crystals is the goal that has been pursued in the development of protein crystallography.

In the protein crystallization process, the first step is to reach supersaturation, which has a decisive impact on the nucleation and growth of protein crystals, further affecting the morphology, quality, and size distribution of crystals [7,8]. The nucleation and growth of protein crystals are complex processes that can be influenced by many factors, including the buffer solution, pH [9], temperature [10,11], precipitant, and protein concentration [12]. In particular, precipitants, which can be mainly divided into inorganic salts and organic polymers, are of great importance [13]. Different precipitants have different mechanisms to promote protein crystallization. Inorganic salt precipitants can destroy the hydration layer of proteins, thereby reducing the binding capacity of proteins to water and increasing the binding capacity between proteins. In contrast, organic precipitants enhance the electrostatic repulsion and polarity between protein molecules by reducing the dielectric constant [14]. The concentration of precipitant can directly affect the supersaturation of the solution. When the concentration of precipitant is high, the solution is in the nucleation zone or even the precipitation zone. In contrast, a low precipitant concentration results in long crystallization induction time, less crystal nucleation, and slow growth rate [15]. In order to obtain perfect crystals, scientists have also explored various crystallization methods, such as the use of microgravity [16] or an electromagnetic field [17,18]. Lately, new crystallization methods such as in vivo protein crystallization [19], counter diffusion technology [20], supercritical fluid crystallization [21], microgravity-controlled precipitation [22], and agarose gel solution crystallization have been developed to prepare protein crystals [23,24]. Additionally, researchers have also developed a series of fluid manipulation technologies and equipment, providing effective and reliable solutions for high-quality protein crystallization screening [25]. All these developments have greatly promoted the recognition of the three-dimensional structure of proteins, which plays a very important role in the research of small-molecule drugs targeting proteins and promoting the development of protein crystallography [26].

In this review, we introduce the development of protein crystallization from the aspects of nucleation mechanisms, heterogeneous nucleating agents, the development of crystallization strategies, and applications in X-ray diffraction and pharmaceuticals. Finally, the bottleneck of protein crystallization and the prospect of future technology development are reviewed.

## 2. Protein Crystallization Nucleation

### 2.1. Homogeneous Nucleation

Nucleation is known as a key step to determine protein crystallization [27,28]. The classical nucleation theory is the most widely used theory to describe the nucleation process. A fluctuation in solution concentration enables the formation of reversible and droplet-like molecular clusters, which develop into thermodynamically stable crystal nuclei and then grow into crystals [29]. The classical theory is typically used to describe single component nucleation, whereas it shows limitations in binary or multicomponent nucleation [30].

**Figure 1:**
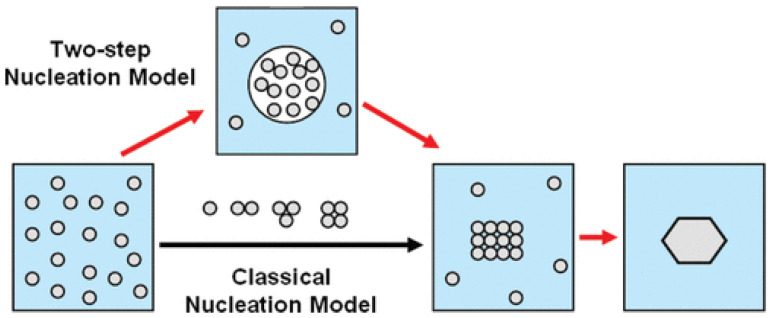
Classical and two-step nucleation models. Reprinted from [32] with permission.

Wolde and Frenkel reported a two-step mechanism, which exhibited a metastable intermediate phase before the formation of the final crystal structure [31]. A solute molecular cluster of sufficient size was first formed, followed by reorganizing into metastable mesophase, where the nucleation takes place [29]. Protein molecules form a crystal nucleus, which will serve as a structural template to guide the orderly arrangement of other molecules (Figure 1) [32]. This is supported by both experimental results and theoretical simulations. Although the two-step nucleation theory was initially proposed to illustrate the process of protein nucleation and crystallization, recent experimental and theoretical studies show that the theory is also applicable to elucidate the nucleation and crystallization of macromolecules and organic small molecules. For example, Sun et al. demonstrated a two-step strategy to construct supramolecular structures, which mimics the multiple pathways of protein crystallization [33,34].

### 2.2. Heterogeneous Nucleation

In addition to the above two crystallization theories, heterogeneous nucleation theory also plays an important role in protein crystallization. In 1988, heterogeneous nucleation was first reported as a nucleating manner for protein crystallization [35]. Heterogeneous nucleation can be considered surface- or particle-assisted nucleation [36]. In this process, supersaturation is typically not enough to achieve homogeneous nucleation [29]. This theory proposes that heterogeneous nucleating agents interact with protein molecules and then produce a higher local active protein concentration, which is conducive to the formation of pre-nucleation clusters [37]. Heterogeneous nucleating agents can stabilize these clusters and promote further growth [27,38].

## 3. Heterogeneous Nucleating Agents

### 3.1. Nucleating Agents from Natural Sources

**Figure 2:**
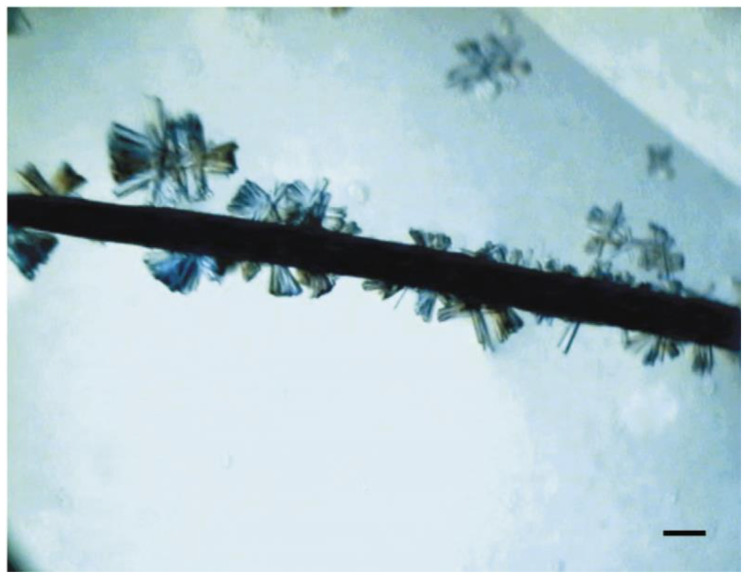
Crystals of potato serine-protease inhibitor growing on a hair fiber. Reprinted from [7] with permission. The scale bar corresponds to 100 mm.

Many reports have shown that proteins can nucleate on contaminants such as dust and fibers in crystal droplets. Hair, especially horse hair, has also been used to promote protein crystallization, which is an ideal choice for capturing protein molecules due to its sharp microstructure and overlapping cuticle (Figure 2) [7]. Experimental results show that horse hair can effectively promote the crystallization of three proteins, including Fab-D protein. In addition, human hair can also promote efficient crystallization of the potato serine protein inhibitor that is typically very difficult to crystallize. Thakur et al. tested 9 natural nucleating agents for 10 model proteins and demonstrated that dried seaweed powder can effectively promote protein crystallization [39]. In addition, cellulose and hydroxyapatite powder also have a nucleation effect, but not as obvious as the seaweed. Another interesting phenomenon is that these natural nucleating agents can also inhibit the crystallization of other proteins while promoting the crystallization of specific proteins. Natural minerals can also promote protein crystallization [7]. In 1988, Mcpherson and Paul used 15 different minerals as nucleating agents to conduct crystallization experiments of four model proteins, including canavalin, concanavalin B, beef liver catalase, and lysozyme. They showed that minerals can effectively promote protein nucleation and crystal growth [40].

### 3.2. Short Peptide Supramolecular Hydrogels

**Figure 3:**
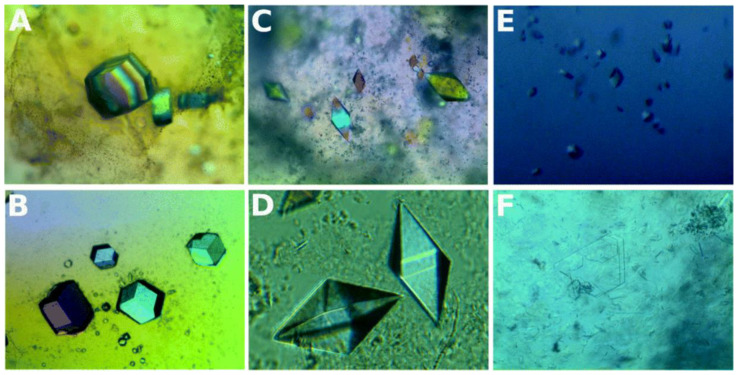
Crystals of lysozyme (**A**), glucose isomerase (**B**), thaumatin (**C**,**D**), insulin (**E**), and FASE (**F**) grown in hydrogels. Reprinted from [43] with permission.

At present, supramolecular hydrogels can be used as nonconvection media to grow high-quality protein crystals [41,42]. Such supramolecular hydrogels have been widely used in the biomedical field due to their biocompatibility [43]. In particular, short peptide hydrogels are known to form a well-defined 3D ordered structure in stereochemistry [44]. Cienfuegos et al. used the intrinsic chirality of short peptides that can interact with protein diastereomers [45]. This makes short peptide supramolecular hydrogel a good medium for obtaining high-quality protein crystals (Figure 3) [43]. Therefore, short peptide hydrogels have received extensive attention in protein crystallization [46]. They have the potential to manipulate protein solubility without affecting the protein structure or biochemical properties. Although several factors can affect protein crystallization, solubility is a key and relatively atypical factor. In particular, short peptide supramolecular hydrogels can stabilize insulin crystals to a higher degree and slow their release [47,48].

### 3.3. DNA

**Figure 4:**
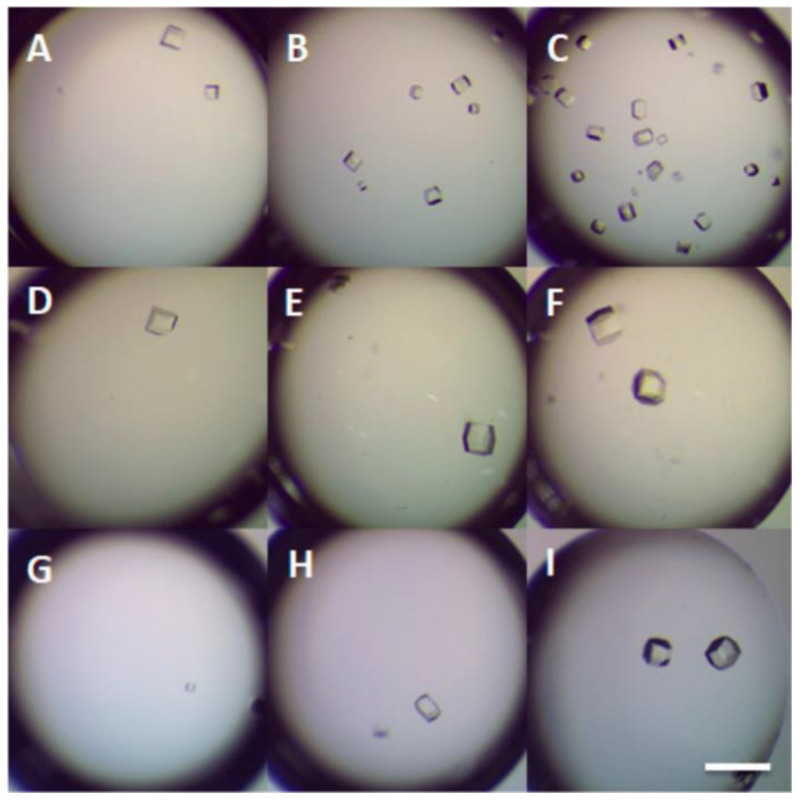
Crystal pictures of 10 mg/mL lysozyme crystallization for 24 h using (**A**) 0 mg/mL calf DNA, (**B**) 1.0 mg/mL calf DNA, (**C**) 5.0 mg/mL calf DNA, (**D**) 0 mg/mL salmon DNA, (**E**) 10 mg/mL salmon DNA, (**F**) 20 mg/ mL salmon DNA, (**G**) 0 mg/mL herring DNA, (**H**) 10 mg/mL herring DNA, and (**I**) 20 mg/mL herring DNA. Scale bar: 600 µm. Reprinted from [52] with permission.

A large number of experiments have proved that DNA can be used to promote protein crystallization and may be applied to proteins with difficulty in crystallization. Seeman et al. proposed a few decades ago that protein crystallization can be realized by a series of molecules arranged in the highly ordered structure of DNA building blocks [49,50]. Heng et al. proposed for the first time that DNA origami can be used as a seed to promote protein crystallization [51]. The size and shape of DNA origami are precisely controlled through programmable characteristics and accurate recognition, and the performance of this material is completely consistent. The existence of DNA origami improves the possibility of low-concentration protein crystallization. DNA was found to shorten the induction time of protein crystallization and increase the number of crystals per drop (Figure 4) [52]. In Figure 4, the samples (B, C) with calf DNA have the largest number of crystals per drop, whereas the samples with salmon and herring DNA form larger crystals (D–I). In addition, it was shown that DNA as a heterogeneous nucleating agent could also effectively improve the crystallization rate and control the crystal size. In general, DNA, as a new polymer additive, promotes protein crystallization and greatly improves the success rate of low-concentration protein crystallization. Considering the programmable and designable characteristics of DNA, specific DNA with a clear sequence and length can be synthesized. Therefore, DNA is expected to have excellent potential for improving systems where biomolecules are difficult to crystallize, thus making maximum use of scarce resources [53].

### 3.4. Nanoparticles

**Figure 5:**
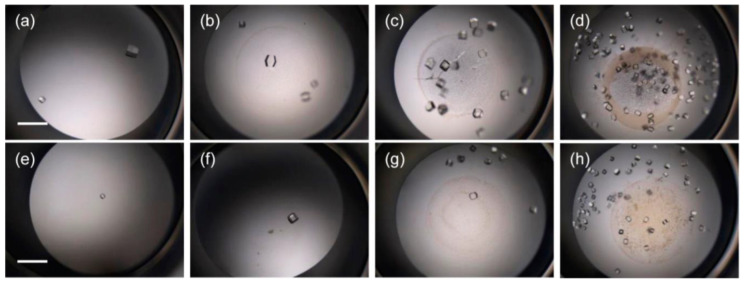
Lysozyme (20 mg/mL) crystallization in the presence of 100 nm ND films (**a**–**d**) and 30 nm ND films (**e**–**h**) at concentrations of 0, 50, 250, and 500 μg/mL. Scale bar: 500 μm. Reprinted from [59] with permission.

**Figure 6:**
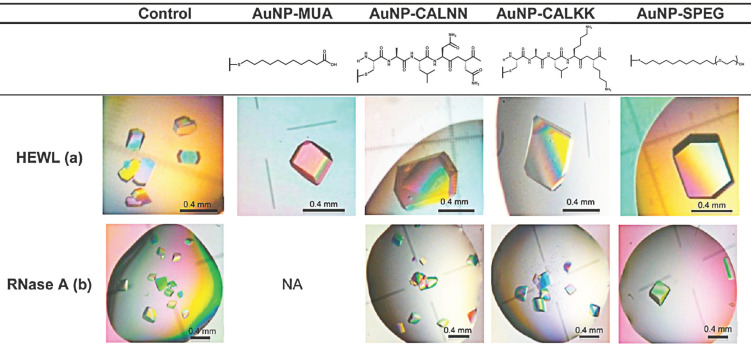
Proteins incubated in the presence of 3 nM AuNPs solutions with (**a**) 25 mg/mL in 50 mM CH_3_COONa and pH 4.5 + 5% NaCl, (**b**) 50 mg/mL in 50 mM CH_3_COONa and pH 5.5 + 3 M NaCl + 1.2 M (NH4)_2_SO_4_. Reprinted from [61] with permission.

Nanoparticles have a large adsorption surface area, which improves the possibility of binding with protein molecules. In addition, nanoparticles can effectively reduce the nuclear barrier, increase the amount of protein nucleation, and thus promote protein crystallization [54,55,56,57]. It has been reported that nanomaterials in the form of aggregates or films could improve the crystallization efficiency of all proteins [58,59]. Nanodiamond (ND) is a kind of carbon-based nanomaterial that has extensive biological application potential [60]. One such application is to promote the nucleation of protein crystals in aqueous solutions (Figure 5) [59]. Through lysozyme, ribonuclease A, proteinase K, and catalase tests, it was found that ND with smaller particle size can adsorb protein more efficiently. Gold nanoparticles (AuNPs) in particular have unique properties, as gold occupies a unique position in the periodic table of elements [47]. Its chemical properties are stable, and it has unique optical properties. Nanoscale gold has better properties than other metals and shows a unique structure and electronic, magnetic, optical, and catalytic properties. This makes it a very attractive material for developing biological nanosystems. Carvalho and Franco et al. concluded from systematic tests and subsequent observation that the introduction of AuNPs should be explicitly considered in the crystal optimization test to improve the previously determined crystallization conditions (Figure 6) [61]. For many proteins that are difficult to crystallize, such as phenylalanine hydroxylase (PHA), myoglobin, native aldehyde oxidase (AOH), its mutant AOH-Y885 M, and albumin, AuNP showed good ability to induce crystallization and the obtained protein crystals possessed good diffraction. Based on the coupling of AuNPs with biomacromolecules and their wide application and interesting interactions in biomaterials, AuNPs may become potential reagents in protein crystallization experiments [61].

### 3.5. Ionic Liquids

**Figure 7:**
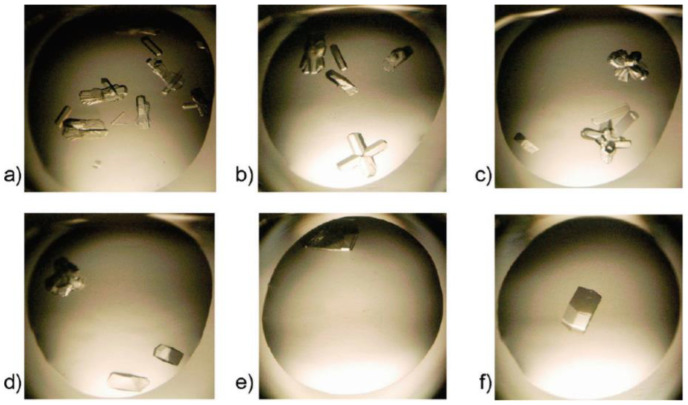
Lysozyme crystallization promoted in the presence of (**a**) 6%, (**b**) 9%, (**c**) 12%, (**d**) 15%, (**e**) 18%, and (**f**) 21% (*w*/*v*) of 1-ethyl-3-methylimidazolium chloride. Reprinted from [64] with permission.

Ionic liquids (ILs) are liquids composed of positive (cationic) and negative (anionic) charges combined by electrostatic interaction [62]. The electromigration of ionic liquids is usually lower than that of the corresponding free ions in aqueous solutions, but it is still too high to observe molecular packing in crystalline solid salts [63]. Therefore, ILs can be regarded as a charged space, which does not form a regular structure, but still maintains a close relationship. Ionic liquids are ideal solvents for biomaterials because of their various properties. Ionic liquids cause changes in crystal morphology and in some cases promote significant increases in crystal size. Crystals grown by Judge et al., using ionic liquids as precipitants or additives, provided a similar or better X-ray diffraction resolution than crystals obtained without ionic liquids (Figure 7) [64]. ILs have been widely used as additives for protein crystallization. Free ionic monomers in ionic liquid solvents provide the possibility to regulate specific interactions, especially anionic hydrogen bonds and cationic surfactant effects. Protein solubility is affected by the action of ionic liquids, inducing protein precipitation and crystallization [65]. Since ionic liquids can slow down the vapor transport rate and control the crystal growth rate, the influence of ionic liquids on the crystallization process is even more obvious than that of precipitation [66].

### 3.6. Porous Materials

**Figure 8:**
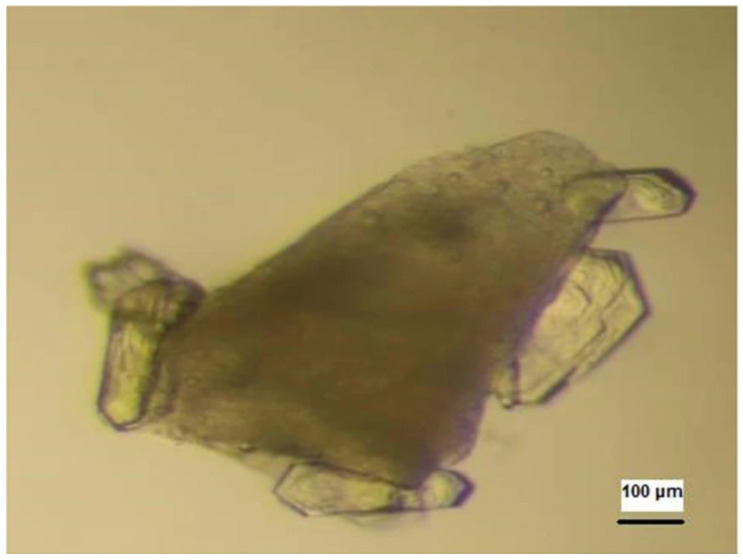
Crystals of a beta lactamase growing on bioglass. Reprinted from [71] with permission.

Porous materials can adsorb protein molecules, which are liable to arrange in crystal order. Large single crystals have been reported by using porous materials without sufficient spontaneous nucleation [67]. The holes in porous materials will capture the protein molecules, and the combined diffusion adsorption action can increase the concentration of protein in the holes [68]. It can also enable the crystals to nucleate, which promotes the formation of crystals, thus improving the crystallization rate and quality. Porous silicon is the first reported porous material to promote protein crystallization [69]. Zhang et al. developed a type of CaO-P_2_O_5_-SiO_2_, an amorphous mesoporous bioactive gel glass with pore size distribution in the range of 2–10 nm in diameter. They demonstrated that the obtained bioactive gel glass can effectively promote protein crystallization [70]. Bioglass has been reported to succeed in producing high-quality crystals of model proteins and target proteins. Nanev et al. reported that bioglass promoted the crystallization of 14 proteins, the highest number of mononuclear reagents known. Moreover, most of these proteins are difficult to crystallize under normal conditions (Figure 8) [71].

**Figure 9:**
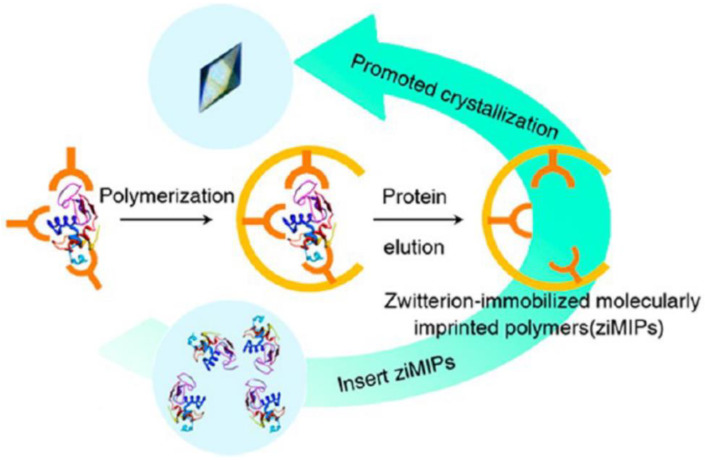
Schematic diagram of zwitterion-immobilized molecularly imprinted polymer synthesis. Reprinted from [73] with permission.

In particular, molecularly imprinted polymer (MIPs), also known as “smart materials”, employ molecular self-assembly to create cavities that can rebind the corresponding proteins [72]. Such unique properties enable MIPs to serve as ideal templates for crystal formation. Ren et al. successfully fixed the zwitterion on molecularly imprinted polymers and obtained zwitterion-immobilized molecularly imprinted polymers (ziMIPs). ZiMIPs could effectively improve the crystal quality of lysozyme, trypsin, catalase, proteinase K, concanavalin A-IV, and somatine, and greatly shorten the crystallization time (Figure 9) [73]. Chayen et al. used six different molecularly imprinted polymers to promote the crystallization of nine different proteins [74]. No crystals were generated without the addition of molecularly imprinted polymers.

## 4. Crystallization Strategies

### 4.1. Functional Interfaces

**Figure 10:**
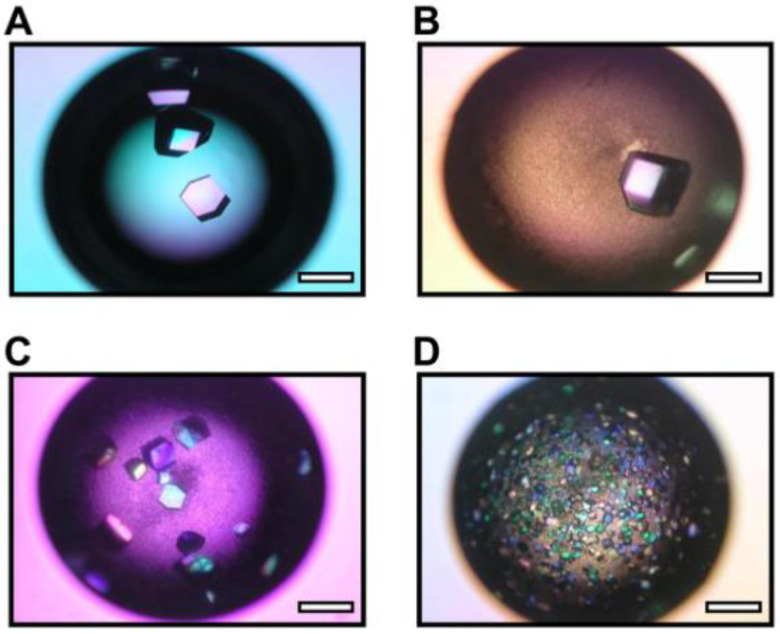
Micrographs of lysozyme crystals in the absence of layered silicate (Control) (**A**) and in the presence of sericite (**B**), K-tetrasilicic fluoromica (**C**), and Na-tetrasilicic fluoromica (**D**). Scale bar: 0.5 mm. Reprinted from [76] with permission.

The crystallization of most proteins begins via heterogeneous nucleation. In fact, the crystallization process usually occurs at the solid interface present in the solution [53]. The solid surface provides nucleation sites such that the nucleation potential barrier on the surface is lower than that in the bulk solution. Different types of solid surfaces exhibit different surface energies, and the nucleation barrier depends on the characteristics of the solid surface [75]. Therefore, treatment of the solid surface may change the surface properties to increase the chances of obtaining protein crystals. Solid surfaces with relatively large sizes (smaller particles) were also tested as effective heterogeneous nuclei (Figure 10) [76]. A mineral matrix [40], silylated mica surface [77], lipid bilayer deposited on a glass cover sheet (for membrane protein crystallization) [78,79], a polymer film containing a poly-L-lysine or poly-l-aspartate ionizable group [80], and modified surfaces with different roughness have all been proven to contribute to protein crystallization [81,82]. These processes depend on the electrostatic interaction between the charged surface and surface proteins with the opposite sign net charge [83]. If the surface can be directly used as a crystal plate or glass cover sheet, the additional step of adding nuclei can be avoided, and heterogeneous nucleation can be more easily applied to high-throughput protein crystallization [84,85].

### 4.2. Electricity and Magnetic Fields

**Figure 11:**
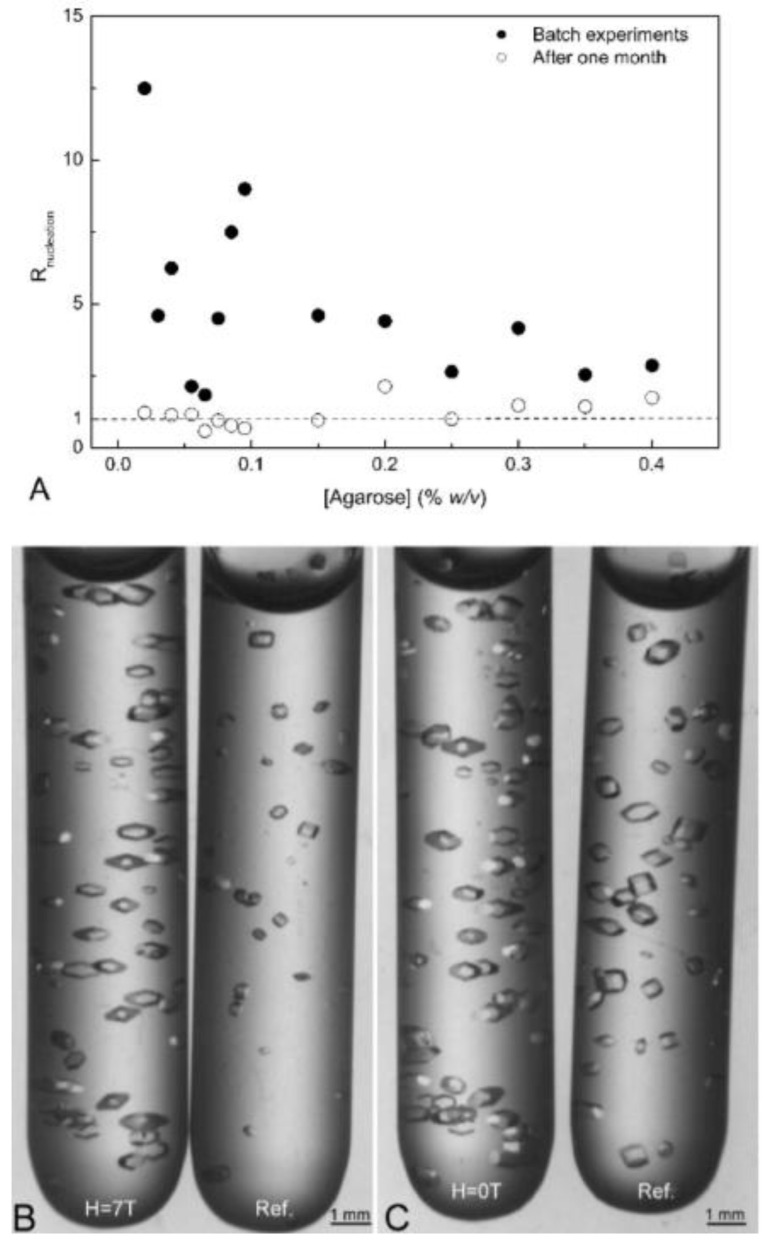
Effects of magnetic field intensity on lysozyme crystallization. (**A**) The number of crystals in magnetic field divided by the number of crystals in the references. (**B**,**C**) Crystals in 0.055% *w*/*v* agarose just after being removed from the magnet. Reprinted from [87] with permission.

Rothgeb and Oldfield first observed the orientation of myoglobin and cobalt myoglobin microcrystal suspensions in the direction of an applied magnetic field in 1981 [86]. Gavira et al. found that the uniform and constant magnetic field shortened the nucleation induction time, resulting in higher nucleation density, larger crystal size, and improved crystal quality (Figure 11) [87,88]. Experimental studies on protein crystallization using high magnetic fields showed that the number of crystal nuclei decreases, the magnetic orientation of microcrystals decreases, the crystal growth rate is relatively slow, and the crystal dissolution rate decreases when compared with those under normal gravity conditions outside the magnetic field [89,90]. Song et al. developed a small and portable device using a 200 mT magnetic field, which improved the nucleation rate, ensured the growth of large single crystals in a short time, and promoted the crystallization of various proteins [91].

**Figure 12:**
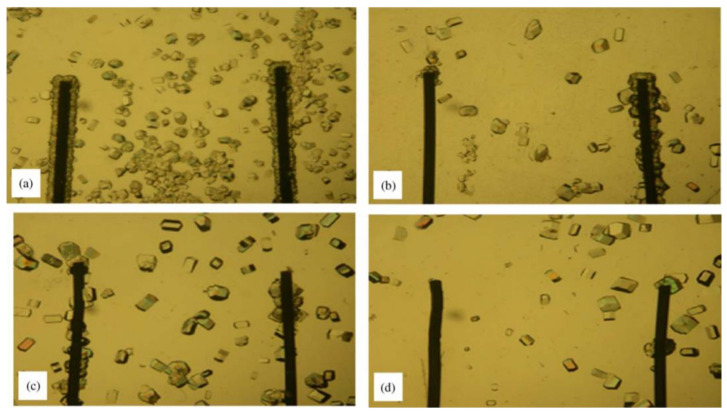
Electric field promotes protein crystallization. Experiments in solution (**a**) without current, (**b**) with current, (**c**) growth in gels without current, and (**d**) growth in gels in the presence of a constant current. Reprinted from [93] with permission.

In addition, an electric field can also promote the formation of protein crystals [92]. A large number of experiments showed that lysozyme crystals only appear around the cathode (negatively charged electrode), while amorphous precipitates are observed near the anode (positively charged electrode) (Figure 12) [93]. By applying a direct current, the number of crystals deposited is significantly reduced, and thus, the size of the crystals is increased. A direct current also shortens the nucleation induction time. For example, using the droplet technique developed in Aubry and colleagues’ laboratory, it has been proved that the external electric field inhibits the nucleation of HEWL crystals, thereby improving the growth rate of lysozyme crystals in the external electric field [94]. The effect of an external electric field and an ultrasonic field on lysozyme crystallization was evaluated by the batch method. It was also observed that the directional growth of the crystals followed a preferential direction toward the cathode [95]. The application of an electric field will produce a small number of large crystals [96]. Crystals grow on the surface of droplets near the cathode. The nucleation rate is greatly reduced, and this experimental method can be used to control the number of crystals in the droplet [97].

### 4.3. Ultrasonic Field

**Figure 13:**
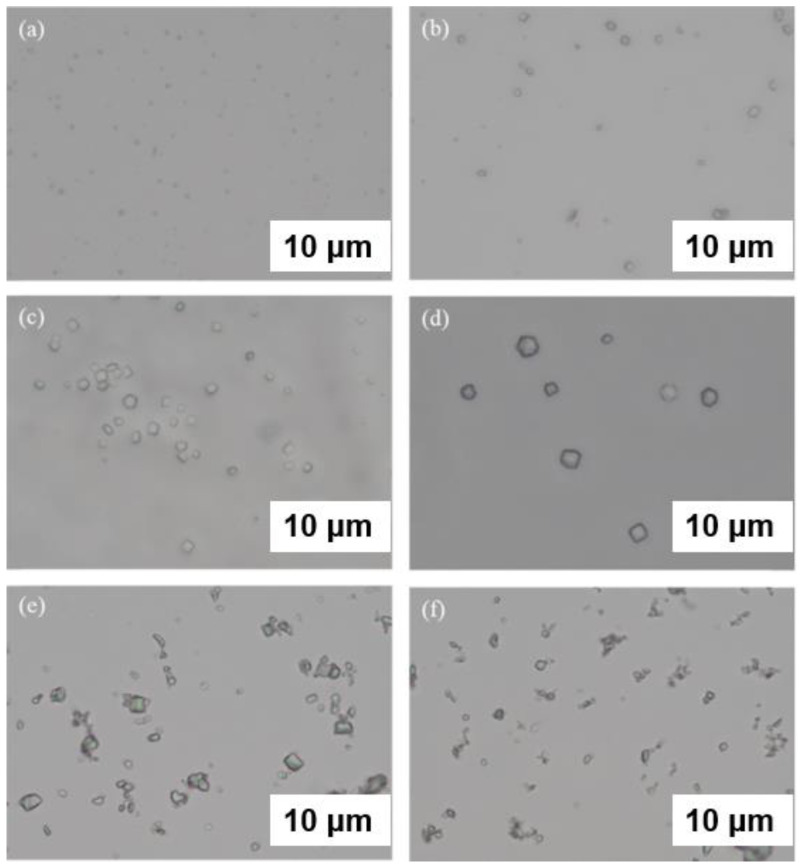
Microscopy images for lysozyme crystals in the nucleation and growth regions (**a**) in the nucleation region of the ultrasound group at ci = 70 g/g buffer solution, (**b**) in the nucleation region of the ultrasound group at ci = 80 g/g buffer solution, (**c**) in the growth region of the ultrasound group at ci = 70 g/g buffer solution, (**d**) in the growth region of the ultrasound group at ci = 80 g/g buffer solution, (**e**) in the growth region of the control group at ci = 70 g/g buffer solution, and (**f**) in the growth region of the control group at ci = 80 g/g buffer solution. Reprinted from [100] with permission.

Ultrasonic crystallization has been widely used in many fields, such as pharmaceutical, chemical, and food applications [98]. In the application of food science in particular, high-intensity ultrasound has been explored as a means to improve the crystallization behavior of fat [99]. The effect of ultrasound on primary nucleation and secondary nucleation may be due to a process called cavitation. Ultrasonic cavitation can be defined as the formation of a vapor cavity or bubble in response to an ultrasonic pressure field. The bubbles generated in the process of cavitation can oscillate around their equilibrium position (stable cavitation) or collapse to form a new bubble group (inertial cavitation). The bubbles generated by cavitation can be used as nucleation sites to induce primary nucleation. On the other hand, if crystals are present before the ultrasonic wave is applied, the high shear force generated during the ultrasonic treatment may induce secondary nucleation through cavitation. Ultrasound can promote the formation of a variety of stable crystal forms in lipid materials and control the polymorphs of crystals. Hao et al. studied the effect of ultrasound on lysozyme crystallization. They demonstrated that under the effect of an ultrasound field, the induction time was significantly shortened, and the aggregation of protein molecules was reduced, which promoted nucleation and increased the crystal size (Figure 13) [100]. Martins et al. also showed that an ultrasonic field could facilitate protein nucleation and improve the quality of protein crystals, which resulted in improved diffraction performance [101]. However, in other research by Hao et al., it was shown that the energy of the ultrasonic field could denature the protein and inhibit protein crystallization once the ultrasonic field was performed for a long time [102].

## 5. Applications of Protein Crystallization

### 5.1. X-ray Crystallography

**Figure 14:**
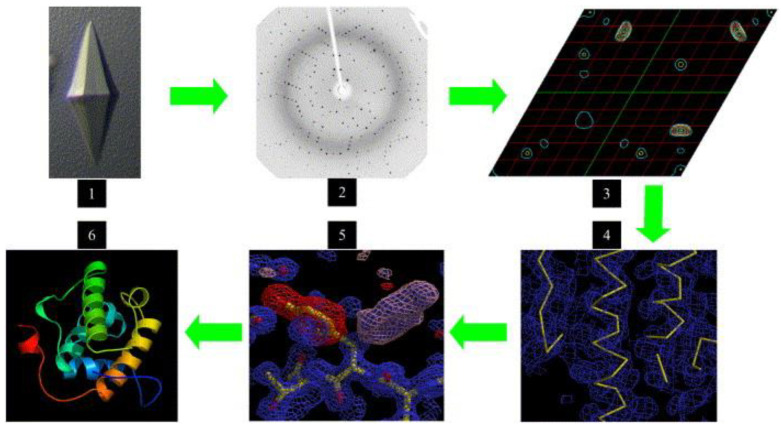
A refinement–analysis–adjustment–refinement cycle to generate the protein model (6). (1) single crystals, (2) oscillation images, (3) a Patterson map, (4) an initial trace of the model, (5) multiple cycles of validation, model re-fitting, and refinement. Reprinted from [104] with permission.

Protein crystals can be used not only for protein purification but also for the determination of protein structure by X-ray diffraction [103]. X-ray crystallography is the primary means and the most important technology to obtain the atomic resolution of a protein structure. The XRD data of protein crystals are analyzed, calculated, and simulated to achieve the protein model (Figure 14) [104]. Obtaining high-quality protein crystals has always been the bottleneck of X-ray single-crystal diffraction technology [105]. The addition of heterogeneous nucleating agents can promote the formation of well-defined crystals, which facilitate improved X-ray diffraction results. As a standard technology in biochemistry and molecular biology, X-ray crystallography has made great progress in the past two decades [106]. At present, X-ray crystallography is becoming a source of information that can not only explain the structure of proteins but also predict the biological characteristics of proteins [107,108].

### 5.2. Pharmaceuticals

**Figure 15:**
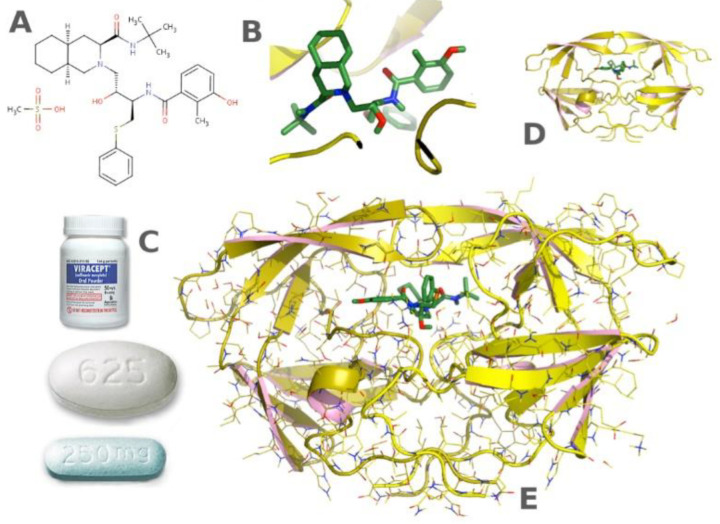
Application of the protein crystallization in pharmaceutics. (**A**,**B**,**D**,**E**) Structure-based drug design of HIV protease inhibitors. (**C**) The formulation for the patient. Reprinted from [113] with permission.

Protein crystallization technology is widely used in the biological pharmacy and food industries [109]. Crystallization is typically the last step in many industrial processes used to produce drugs [110]. The function of a protein is closely related to its three-dimensional structure, which plays a very important role in the research of small-molecule drugs targeting certain proteins [111,112]. With more and more protein structures being determined, structure-guided drug design has become an important method for many companies to develop excellent candidate drugs (Figure 15). Agouron Pharmaceuticals has developed Nelfinavir through structural analysis of protein, which is a key component of antiretroviral therapy for AIDS [113].

## 6. Discussion and Perspectives

With the increasing demand for biological drugs in the market, macromolecular drugs such as proteins have attracted more and more attention due to their unique functions. A therapeutic protein in crystalline form has many advantages over its solution form, including higher stability, higher dose concentration, and better release control, such as insulin, infliximab, and trastuzumab. Developing a method that can enhance the protein crystallization process is key to the successful development and large-scale production of protein crystallization drugs. Due to the characteristics of protein such as large molecular weight and high molecular flexibility, it is difficult to obtain high-quality protein crystals. Heterogeneous nucleating agents can reduce the potential barrier to nucleation and make protein crystallization easier. The protein crystallization process is not only sensitive but also has poor repeatability. The strict control of various conditions will also have an impact due to subtle changes in some external factors, such as temperature, pressure, pH, and other factors. Although many methods have been used to conduct the process of protein crystallization, the crystallization effect is very limited and cannot meet expectations. In protein crystallinity research, most of the studies are only conducted for several commonly used proteins, so when these techniques are used to screen other protein crystallization conditions, they lack universal applicability. To resolve this problem, it is necessary to fully understand the crystallization laws of proteins, combine advanced science and technology, and adopt more crystallization strategies and methods to make the target protein easier to crystallize.

## Data Availability

Not applicable.
